# Adaptation to chronic exposure to sepantronium bromide (YM155), a prototypical survivin suppressant is due to persistent DNA damage-response in breast cancer cells

**DOI:** 10.18632/oncotarget.26096

**Published:** 2018-09-11

**Authors:** Tasaduq H. Wani, Sreeraj Surendran, Vishnu S. Mishra, Jaya Chaturvedi, Goutam Chowdhury, Anindita Chakrabarty

**Affiliations:** ^1^ Department of Life Sciences, Shiv Nadar University, Greater Noida, UP 201314, India; ^2^ Department of Chemistry, Shiv Nadar University, Greater Noida, UP 201314, India

**Keywords:** breast cancer, survivin, YM155, drug-adaptation, DNA damage

## Abstract

Sepantronium bromide (YM155), originally developed against the anti-apoptotic protein survivin, performed exceptionally well in pre-clinical and phase I clinical trials. However, in phase II trials of several cancer types including breast cancer it performed poorly. Additionally, no definitive correlation between survivin level and response to therapy was found. In an attempt to understand the true reason of the late-stage failure of this promising drug, we developed YM155-resistant MCF-7 breast cancer cell line and characterized side-by-side with the drug-naïve parental cell line. Chronic YM155 treatment resulted in downregulation of survivin expression yet triggered cellular responses typical of adaptation to persistent DNA damage. Lowering endogenous antioxidant glutathione level and activity of cell cycle check-point kinase restored YM155 activity. Thus, contrary to its development as a survivin suppressant, YM155 primarily acts as a chemotherapeutic drug causing oxidative stress-mediated DNA damage. Adaptation to long-term exposure to YM155 can be prevented and/or overcome by interfering with detoxification and DNA damage-response pathways. Finally, proteins associated with DNA damage-response pathway will be more appropriate as predictive biomarkers of YM155 in breast tumor cells.

## INTRODUCTION

The major function of the IAP family proteins is suppression of caspases, the effector enzymes for apoptosis [1]. Survivin, the smallest IAP protein, is unique, since it is highly expressed in cancer cells and functions in multiple processes of tumorigenesis [2]. Survivin overexpression in most cancers, including breast cancer (BC) is associated with advanced disease, poor prognosis, therapy resistance and faster recurrences [3]. In multiple FDA-approved genomic tests for BC such as Oncotype Dx, Mammaprint and Prosigna, survivin is used as a predictive and prognostic biomarker [4].

YM155, a small imidazolium compound, identified from a pharmacological screen based on the survivin core promoter inhibition, is the first in the class of drugs called “survivin suppressant” [5]. In pre-clinical studies, YM155 exhibited strong anti-tumor and anti-metastatic activities [6]. In two phase I clinical trials with patients suffering from advanced stages of various solid tumors refractory to standard therapies, YM155 was administered for up to 168 cycles [7, 8]. Based on the positive results, multiple phase II studies were carried out in diverse cancer types. However, in most trials, including one with HER2-negative BC patients, YM155 failed to demonstrate significant anti-tumor efficacy either as a monotherapy or in combination with standard chemotherapy [9–13]. This could partly be explained on the basis of absence of biomarker-based patient selection strategy which demands knowledge of exact mechanism of drug action. For YM155, this is particularly true, since apart from being a survivin suppressant, it has been reported to either inhibit several proteins having anti-apoptotic and growth stimulatory functions [14, 15] or act as a potent DNA-damaging agent [16]. It is also unclear whether downregulation of target protein/s or execution of genotoxicity is necessary and sufficient for its anti-tumor action.

Another explanation for its less than satisfactory clinical activity for most cancer types could be development of adaptive resistance. Understandably, such type of event would require the drug having a generalized function since in some cancer types the intended target will be expressed at higher level, hence conferring greater sensitivity to the drug than others.

In the work described here, we have used a YM155-resistant model in an attempt to gain insight into its primary mode of action in BC cells. Using estrogen receptor (ER)-positive MCF-7 cell line-derived YM155-resistant (YMR) cells, we have shown that chronic YM155 exposure triggers persistent DNA damage-associated adaptive responses while continuing to downregulate survivin at the same efficiency as the drug-naïve cells. Cellular sensitivity to YM155 was restored by interfering with such adaptive mechanisms such as inhibiting endogenous anti-oxidant glutathione levels or cell cycle check-point arrest. Together, we present convincing evidence that although developed as a targeted drug with specificity against survivin, YM155 is primarily a genotoxic agent, at least in ER+ BC cells. Accordingly, molecules associated with DNA damage-response pathway rather than survivin should be considered as its pharmacodynamic biomarkers.

## RESULTS

### Chronic exposure of MCF-7 cells to YM155 induces adaptive drug resistance

**Figure 1:**
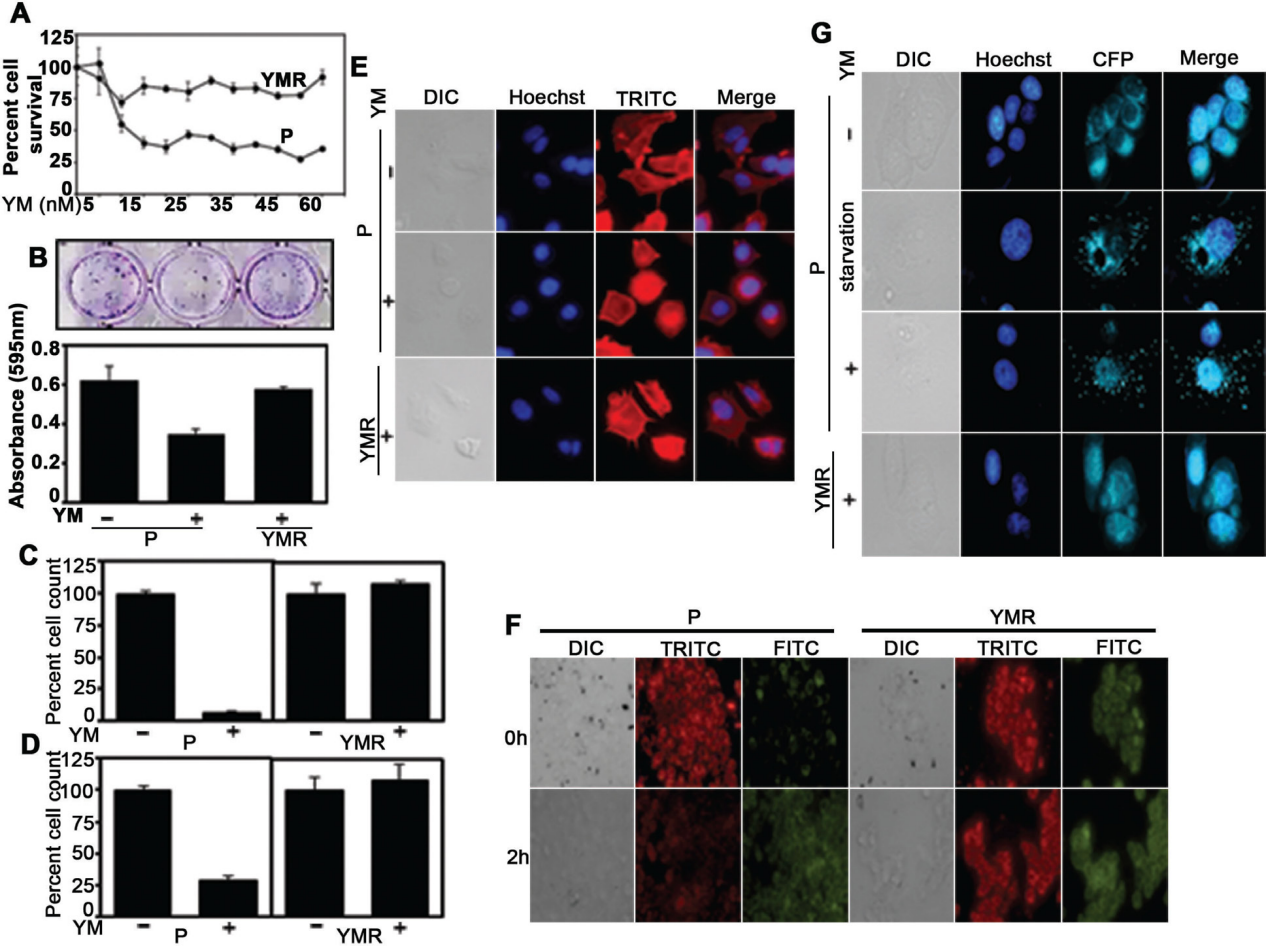
YMR cells remain insensitive to YM155 (**A**) CellTiter-Glo assay comparing viability of P versus YMR cells (plated in equal number) exposed to increasing doses of YM1555 over a 72 h period. (**B**) Top panel. Colony escape assay testing long-term proliferation of P and YMR cells treated with 40 nM YM155 for 72 h. Bottom panel. Quantitation of colonies survived after 10 d YM155 exposure. (**C** and **D**) 3D growth assay testing sensitivity of P versus YMR cells to 40 nM YM155. Cells were grown on **(C**) basement membrane composed of Matrigel and (**D**) soft-agar coated plates for 10 d and 8 d, respectively plus/minus 40nM YM155. At the end of the assay, cells were detached from the matrix and counted by trypan blue exclusion method. (**E**) Comparison of gross morphological features of P, P treated with 40 nM YM155 for 72 h and YMR treated with 40 nM YM155 continuously by nuclear (Hoechst) and actin cytoskeletal protein (TRITC) staining, followed by immunofluorescence microscopy. (**F**) Comparison of change in mitochondrial membrane potential (JC-1 staining) of P versus YMR cells exposed to 40 nM YM155 for up to 2 h. A decrease in red (TRITC) to green (FITC) fluorescence is indicative of membrane depolarization. (**G**) MDC staining to compare autophagy induction in P versus YMR cells following 40 nM YM155 treatment for 72 h. MCF-7 cells were starved overnight and used as positive control to induce autophagy. Hoechst and CFP indicate nucleus and autophagosome, respectively.

We believed that a solution to understand why YM155 failed to provide expected clinical benefit, would be to generate a clinically relevant continuously drug-challenged model system. Accordingly, we exposed MCF-7 cell line to increasing drug concentration (starting from sub-IC_50_ concentration of 7.5 nM) (Supplementary Figure 1A) until it reaches 40 nM at which the cells continued to grow, albeit at a rate slower than the parental cells in drug-free condition (Supplementary Figure 1B). However, as shown by the CellTiter-Glo assay, with increasing concentration of YM155, chronically drug-treated (YMR) cells survived considerably better than the drug-naïve parental (P) cells (Figure 1A), indicating that they have devised an adaptation mechanism. Low density plating following 72 h YM155 treatment resulted in greater number of drug-tolerant colonies over a 10-day period (colony escape assay) from the YMR cells compared to its MCF-7 P counterpart (Figure 1B). Similar to two-dimensional (2D) growth, 3D growth of YMR cells on basement membrane (Figure 1C) and on soft-agar-coated plate (Figure 1D) were also unaffected by 40 nM YM155 compared to the P cells. No gross morphological differences, except for the presence of invadopodia-like structures at the edges of the YMR cells were detectable between 40 nM YM155-treated YMR and P cells, (Figure 1E).

IAP family proteins control intrinsic pathway of apoptosis that involves mitochondrial membrane permeabilization [1]. To identify whether lack of growth inhibition in YMR cells is correlated with absence of cell death, we measured changes in mitochondrial membrane potential by JC-1 staining, a cationic dye that shifts form mitochondria-localized red aggregate to diffused cytoplasmic green monomer formation upon depolarization. As shown in Figure 1F, within 2 h of treatment, 40 nM YM155 triggered mitochondrial membrane permeabilization in P cells, leaving YMR cells unaffected. Of note, under base-line condition, YMR cells displayed already depolarized mitochondria compared to drug-naïve P cells (Figure 1F), indicative of presence of compromised mitochondria upon chronic drug exposure. Because YM155 induces autophagy-dependent cell death in BC cells [17, 18], we checked for formation of autophagic vacuoles using a fluorescent compound, monodansylcadaverine (MDC, used for detection of autophagy induction in cells). YM155 treatment for 72 h induced autophagosome-associated puncta formation in P, but not in YMR cells (Figure 1G). Together, these data indicated that long-term exposure to YM155 provoked adaptive responses in BC cells so that they can no longer be growth-inhibited or induced to undergo cell death even at a high drug concentration.

### YMR cells continue to downregulate survivin in presence of YM155

**Figure 2:**
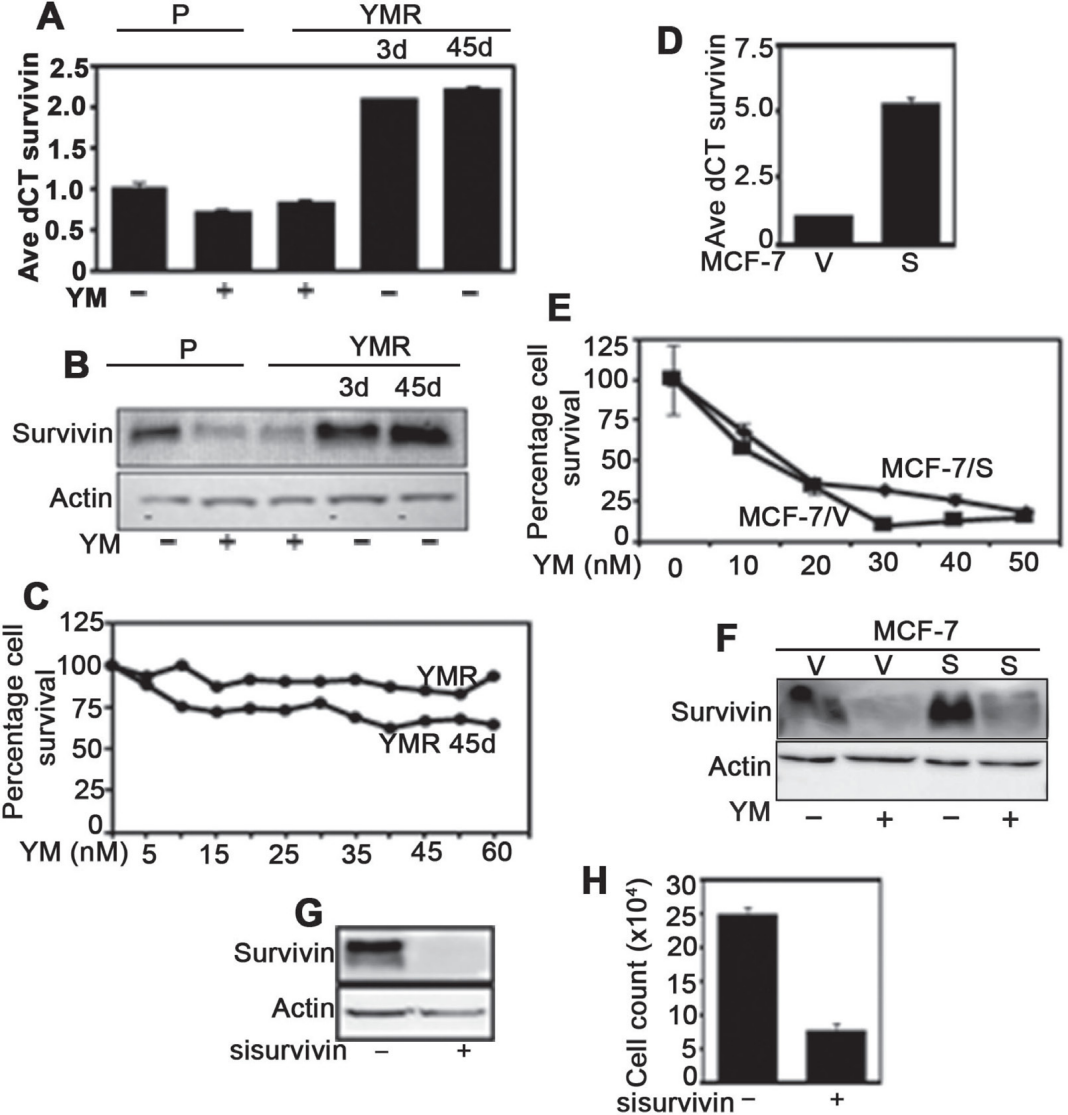
YMR cells downregulate survivin similarly to that of P cells upon exposure to 40 nM YM155 (**A**) Real-time PCR measuring survivin mRNA levels in P cells treated plus/minus 40 nM YM155 (for 72 h), YMR cells continuously exposed to 40 nM YM155, YMR cells starved of YM155 for 3 d and 45 d. (**B**) Immunoblot measuring survivin protein levels in P and YMR cells receiving treatment as mentioned in (**A**). (**C**) CellTiter-Glo assay comparing viability of YMR cells continuously exposed to 40 nM YM155 versus drug starved for 45 d challenged for 72 h with increasing doses of YM155. (**D**) Real-time PCR comparing survivin mRNA levels in MCF-7 cells transfected with empty vector (MCF-7/V) and survivin expression plasmid (MCF-7/S) and selected with G418 for 15 d. (**E**) Comparison of differences in proliferation with increasing doses of YM155 (72 h) of MCF-7/V and MCF-7/S cells. (**F**) Comparison of survivin protein levels in MCF-7/V versus MCF-7/S cells plus/minus 20 nM YM155. (**G**) Immunoblot demonstrating downregulation of survivin protein expression in ctrl versus survivin siRNA-transfected YMR cells (45d drug starved). (**H**) Identifying differences in 72 h proliferation rate between ctrl and survivin siRNA transfected cells.

Because, anti-cancer drug adaptation can be associated with loss of ability to modulate the drug-target protein [19], it is necessary to test whether YM155 continue to downregulate survivin in YMR cells or not. Real-time PCR and immunoblot experiments revealed comparable levels of survivin mRNA and protein downregulation in both P and YMR cells upon exposure to 40 nM YM155 (Figure 2A, 2B). Interestingly, in YMR cells, within 3-days of drug withdrawal, survivin mRNA and protein levels were restored and increased by approximately two-fold than the drug-naïve P cells (Figure 2A, 2B). Culturing YMR cells for 45-days in absence of drug also resulted in higher than baseline levels of survivin transcript and protein (Figure 2A, 2B). However, these cells continued to be YM155-resistant at a level similar to continuously drug-exposed YMR cells (Figure 2C).

If survivin downregulation is essential for the growth inhibitory effect of YM155, it is anticipated that survivin overexpression will hinder this phenomenon. Accordingly, we overexpressed survivin in MCF-7 cells under pCMV promoter (Figure 2D) and treated with increasing doses of YM155 for 72 h. Contrary to our expectation, survivin-overexpressing MCF-7/S cells were affected equally to the cells expressing empty vector (MCF-7/V), (Figure 2E and 2F). Thus, the anti-tumor effect of YM155 in BC cells may not be solely contributed by its ability to suppress survivin expression.

### YMR cells remain sensitive to survivin downregulation

As part of a drug-adaptation program, cancer cells can lose addiction to a drug target (bypass mechanism) [20]. To determine whether chronic drug treatment has endowed MCF-7 cells with the ability to bypass dependency on survivin for cell survival, YMR cells were transfected with survivin siRNA. As shown in Figure 2G–2H, survivin RNAi inhibited proliferation of YMR cells. Therefore, long-term YM155 exposure does not alleviate survivin-dependency of these cells. Since, YM155 continue to downregulate survivin expression in YMR cells without affecting their viability, it is likely not its major mode of action.

### YMR cells exhibit chronic DNA damage-associated phenotypes

**Figure 3:**
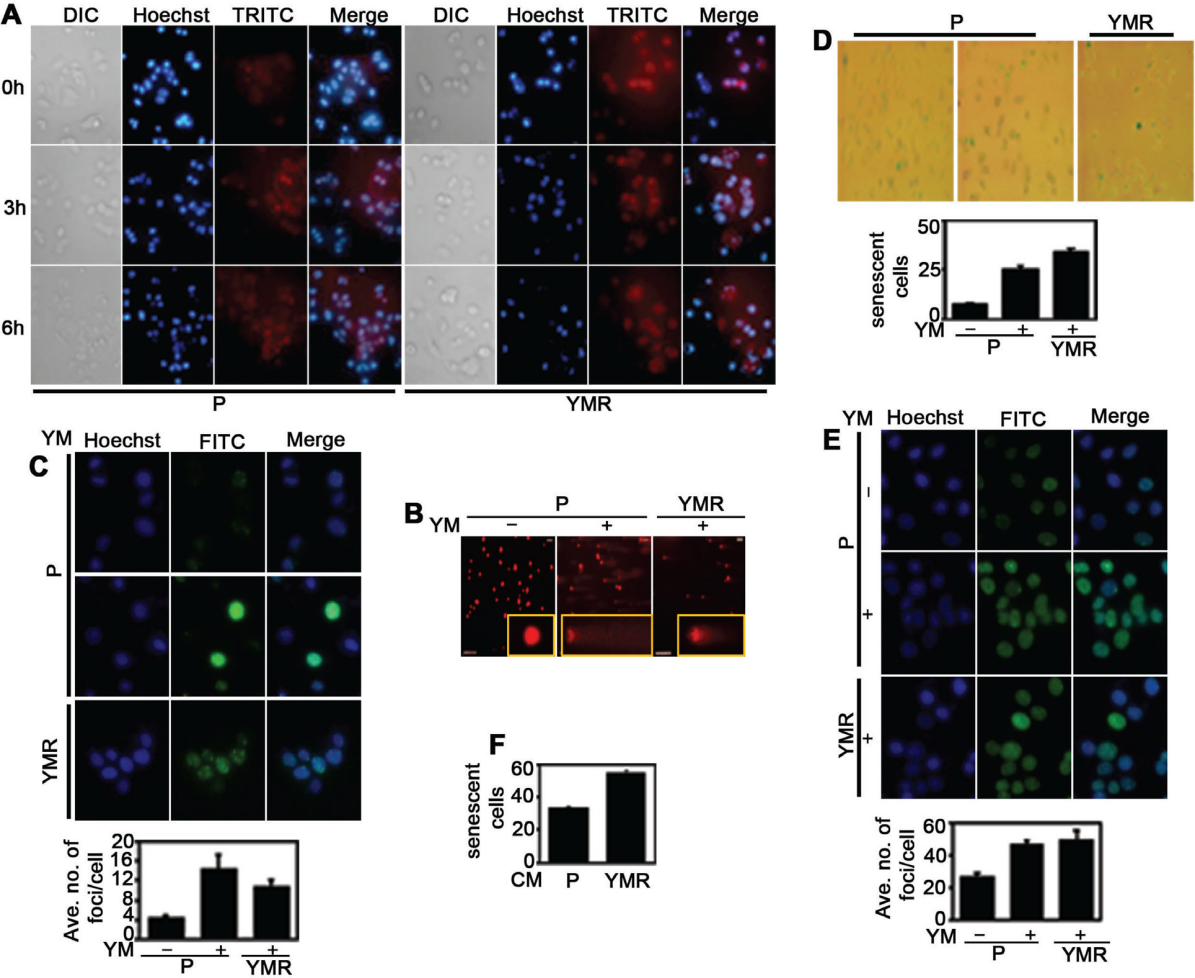
YMR cells undergo persistent DNA damage associated with chronic YM155 treatment (**A**) Measurement of ROS levels following 40 nM YM155 treatment in P versus YMR cells for 3 h and 6 h. Hoechst is nucleus and TRITC is ROS. (**B**) Comet assay demonstrating extent of DNA damage in 40 nM YM155 treated (12 h) P and YMR cells. (**C**) Comparison of γH2AX (FITC) foci formation (top panel: images, bottom panel: quantitation) between P and YMR cells treated with 40 nM YM155 for 72 h. YMR versus P comparison is statistically significant at *p* < 0.05 (0.0007). (**D** and **E**) Assessment of TIS induction as determined by (**D**) SA-βgal immunohistochemistry and (**E**) SAHF (FITC) formation in P and YMR cells exposed to 40 nM YM155 for 72 h. In both (**D** and **E**), bottom panels represent quantitation of the figure from the top panels. YMR versus P comparison is statistically significant at *p* < 0.05 (7.40396E-11: D; 0.0181: E). (**F**) SA-βgal assay comparing senescence induction in MCF-7 cells exposed to CM collected from P and YMR cells for 72 h.

Numerous studies have indicated that YM155 is a potent DNA damaging agent [6, 16]. It is possible that cellular adaptation to this drug is associated with chronic DNA damage-response, a phenomenon often seen with conventional genotoxic agents [21]. First, we decided to check whether YM155 generates reactive oxygen species (ROS) in MCF-7 cells, and if it does so, whether there is difference in ROS level between short versus long-term YM155-exposed cells. Within 3 h of drug addition, ROS production was evident in P cells, which did not further increase at 6h, indicating that YM155 quickly generates oxidative stress inside the cells (Figure 3A, left panel). Comparable ROS level was observed in YMR cells even at 0 h time point, which did not further increase at 3 h and 6 h (Figure 3A, right panel). ROS production was correlated with DNA damage in YM155-treated cells as shown in comet assay. However, the length of the comet tail (indicative of extent of DNA damage) in YMR cells was less compared to drug-treated P cells (Figure 3B). Similar trend was found in S139 phosphorylated histone variant H2AX, γH2AX immunofluorescence foci formation assay (Figure 3C), a marker associated with DNA double-strand breaks (DSBs) [22]. Together, these indicate that similar to P cells, in YMR cells YM155-derived oxidative species cause extensive DNA damage, even though at a lesser extent, possibly due to an adaptation to long-term drug exposure.

Chronic DNA damage by genotoxic agent is often associated with growth arrest, known as therapy-induced cellular senescence (TIS) [23]. We looked at the expression of senescence-associated β galactosidase (SA-βgal) by immunohistochemistry to determine whether continuous exposure to YM155 induces TIS. Indeed, YMR cells demonstrated higher SA-βgal expression, compared to drug-treated P cells (Figure 3D). Trimethylation at Lysine 9 of histone H3 (H3K9me3) is a marker of TIS-associated chromatin modulation (senescence-associated heterochromatin foci/SAHF) [24]. Consistent with SA-βgal positivity, greater numbers of H3K9me3 foci were found in YMR cells compared to drug-naïve P cells. However, the difference was not statistically significant between 72 h drug-treated P and chronically drug-exposed YMR cells (Figure 3E). Another important characteristic of senescent cells is to secrete a plethora of proteins, often known as senescence-associated secretory proteome (SASP), important non-autonomous effectors of senescence [25, 26]. To determine if similar phenomenon is taking place in YMR cells, we collected conditioned media (CM) from serum-starved P and YMR (maintained drug-free for several days) cells, exposed drug-naïve P cells to these two types of CM for 72 h and stained for SA-βgal. Figure 3F clearly demonstrates an increase in number of SA-βgal+ population in P cells exposed to YMR CM, compared to the CM collected from P cells. Collectively, these data indicate that chronic exposure to YM155 induced multiple changes associated with persistent DNA damage in YMR cells including induction of DSB, chromatin modification and TIS.

### YMR cells can be re-sensitized to YM155 by inhibiting cellular antioxidant levels and/or blocking cell cycle checkpoint proteins

**Figure 4:**
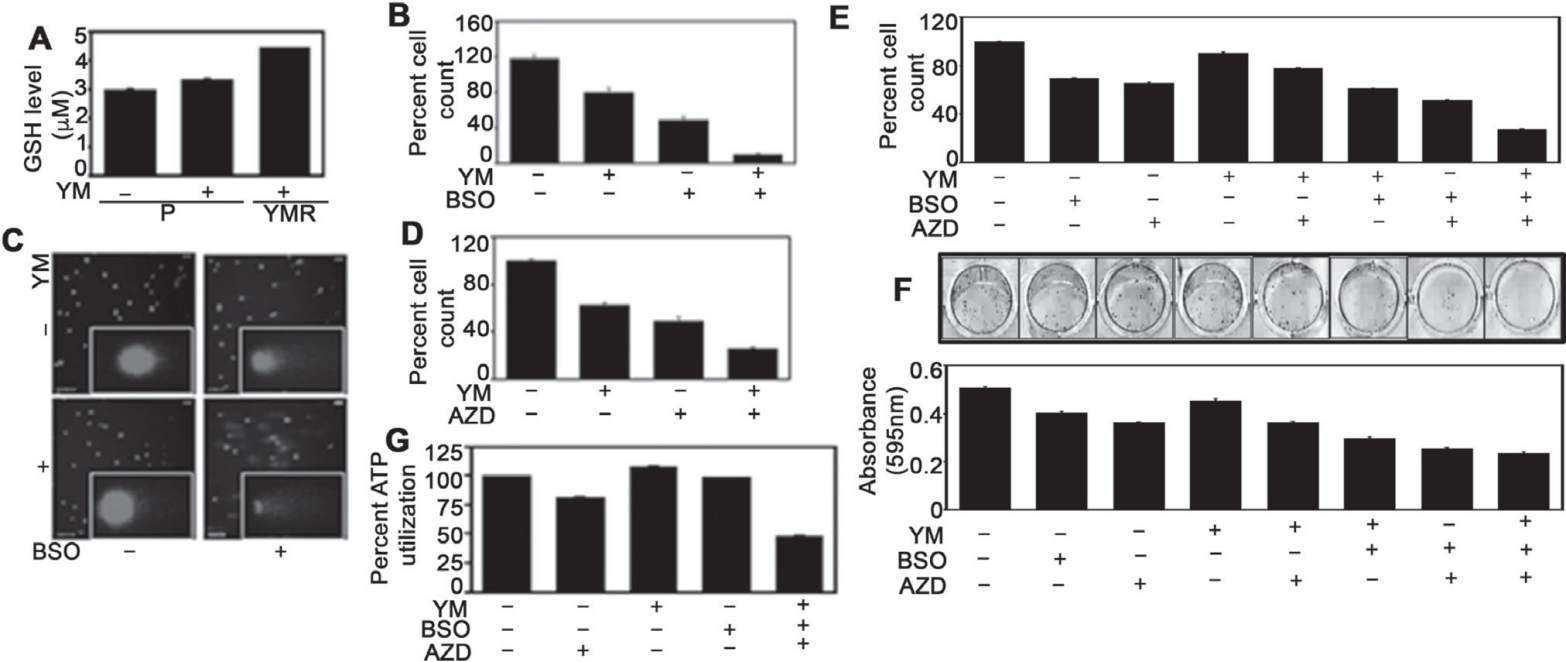
Inhibiting GSH levels and cell cycle check-point arrest restore YM155 sensitivity in YMR cells (**A**) Intracellular GSH measurement in P plus/minus and YMR plus 40 nM YM155 treated (for 72 h) cells. (**B**) Cell counting assay comparing proliferation of P and YMR cells exposed to BSO (including 1 mM pretreatment for 15 h), YM155 (40 nM) and combination of both after 72 h. YM155 versus YM155 + BSO comparison is statistically significant at *p* < 0.05 (0.005166). (**C**) Comet assay assessing DNA damage in cells exposed to treatments mentioned in (**B**). (**D**) Comparison of cell proliferation between P and YMR cells exposed to 50 nM AZD7762, 40 nM YM155 and combination of two after 72 h. YM155 versus YM155+AZD7762 comparison is statistically significant at *p* < 0.05 (0.010199). (**E** and **F**) Comparison of short-term and long-term proliferation by (**E**) cell counting and (**F**) colony escape assays in P and YMR cells treated with BSO **(**including 0.5 mM pretreatment for 15 h), YM155 (40 nM), AZD7762 (50 nM), a combination of two and a combination of three. Statistical analysis indicates that effect of YM versus BSO+YM is significant at *p* < 0.05 (0.0037 in (**E**); 0.0017 in (**F**)) while YM versus AZD+YM remains insignificant (0.0892) in (**E**) and significant (0.0125) in (**F**). Effects of the drugs in double combination AZD+YM or BSO+YM are also significant compared to that of the triple combination BSO+AZD+YM (0.0003 in (**E**) and 0.0017 in (**F**) for AZD+YM; 0.0003 in (**E**) and 0.0080 in (**F**) for BSO+YM, respectively). (**G**) Viability of P and YMR cells treated with BSO **(**including 0.5 mM pretreatment for 15 h), YM155 (40 nM), AZD7762 (50 nM) and a combination of three. YM155 versus YM155+BSO+AZD7762 comparison is statistically significant at *p* < 0.05 (2.78E-05).

In principle, persistent DNA damage due to chronic YM155 exposure may induce adaptive responses. To identify the presence of any such mechanism, we compared the cellular antioxidant glutathione (GSH) levels among drug-naïve P, 72 h drug-treated P and chronically drug-exposed YMR cells. GSH is an evolutionary conserved, abundantly present, endogenous antioxidant that plays important role in preventing damage to cellular components from the harmful effects of oxidative species [27, 28]. Increased GSH levels have been associated with chemoresistance and buthionine sulfoximine (BSO), the irreversible inhibitor of γ-glutamylcysteine ligase (GCL), is the most frequently used agent to experimentally reduce GSH in tumor cells [28]. Although, BC cells in general have higher base-line GSH levels than their normal counterpart [29], further increase in GSH levels was observed gradually from P to P plus drug to YMR cells (Figure 4A). Exposing YMR cells to BSO re-sensitized these cells to YM155 (Figure 4B, Supplementary Figure 2A) which can be correlated with increased levels of DNA damage (Figure 4C).

While protecting cells from harmful effects of drug-induced oxidative species by increasing GSH levels can be seen as the first-line of cellular defence, arresting cells at the cell cycle check-points for repairing damaged DNA resulted from such oxidative species can be considered as a second-line of protective mechanism [30]. Check-point kinase 1 (Chk1) is a serine/threonine kinase primarily responsible for coordinating DNA damage and cell cycle checkpoint responses and its pharmacological inhibition increases sensitivity toward chemotherapeutic agents [31]. Therefore, we combined AZD7762, a potent Chk1 inhibitor [32] with YM155 and followed the proliferation of YMR cells. The combination of two drugs inhibited growth of YMR cells more than each agent alone (Figure 4D, Supplementary Figure 2B). As a pharmacodynamic biomarker we compared pS345 Chk1 levels [33] in P versus YMR cells. Consistent with the literature, pChk1 was increased in AZD7762-treated YMR cells indicating accumulation of damaged DNA (Supplementary Figure 2C). Finally, we combined BSO and AZD7762 to test whether blocking two protective arms against YM155-mediated DNA damage has better inhibitory effect on YMR cells. Simultaneously blocking check-point arrest and lowering GSH levels clearly reversed YM155 resistance as is evident from reduced levels of cell proliferation (Figure 4E, 4F) and viability (Figure 4G).

### ER+ BC cells respond better to YM155 when cellular protective mechanism/s against DNA damage is blocked

**Figure 5:**
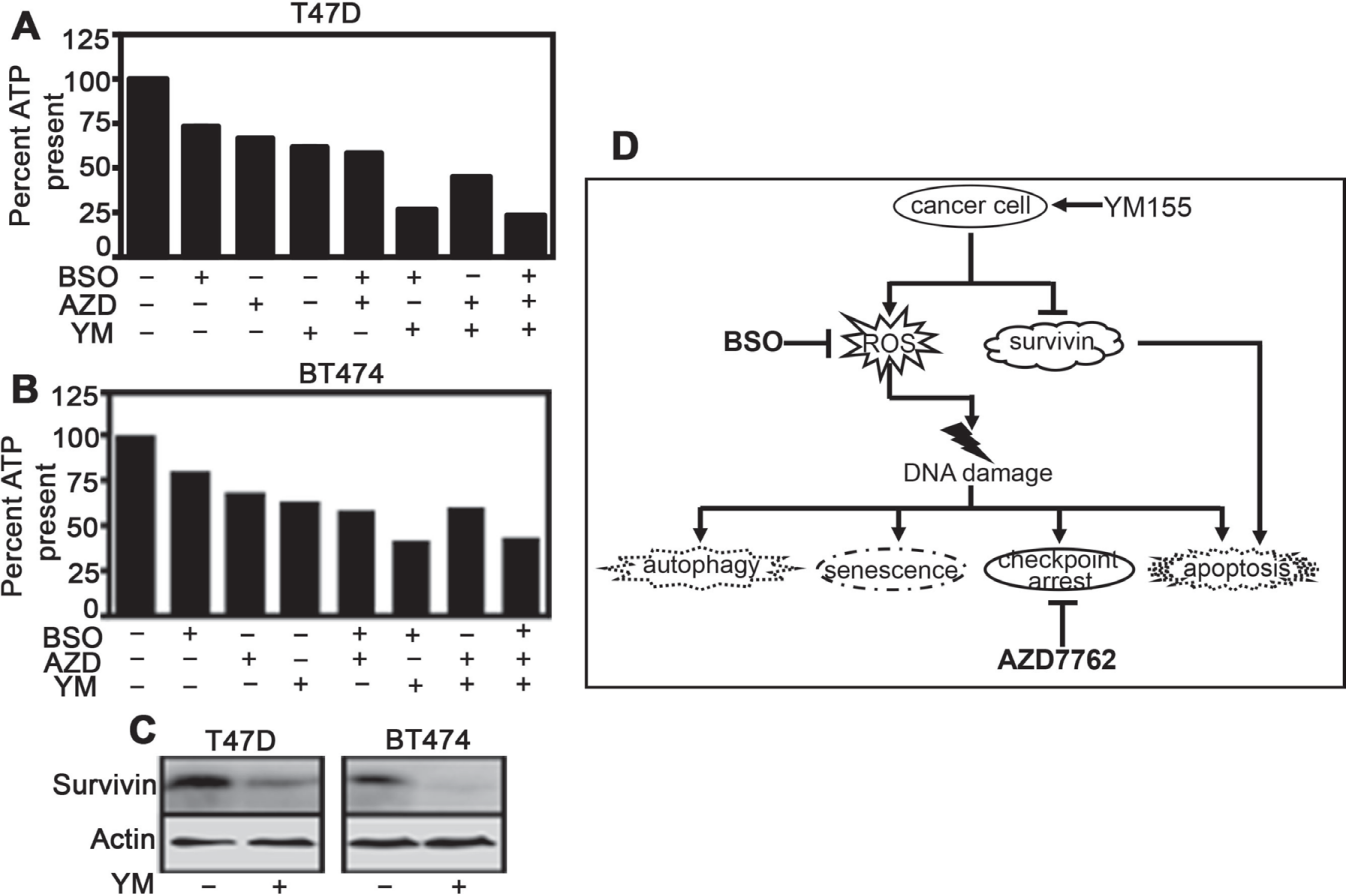
Hindering protective mechanisms against DNA damage renders ER+ cells more sensitive toward YM155 (**A** and **B**) Both cell lines were treated with YM155 (40 nM for T47D and 50 nM for BT474), BSO (including 15 h pretreatment with 0.5 mM), AZD7762 (50 nM) and all three for 72 h and subjected to CellTiter-Glo assay for measurement of cell viability). YM155 versus YM155+BSO+AZD7762 comparison is statistically significant at *P* < 0.05 (2.35411E-07: (**A**); 1.04E-05: (**B**)) (**C**) Immunoblot comparing survivin expression levels in T47D and BT474 following 40 nM and 50 nM YM155 exposure, respectively for 72 h. (**D**) Schematic representation of the mode of action of YM155 on BC cells as established from this study.

If genotoxicity is the primary mode of YM155 action, then combining it with GSH and/or Chk1 inhibitors should enhance its anti-tumor effect in any ER+ BC cells. Accordingly, in two other drug-naïve ER+ BC cell lines BT474 and T47D, combination of AZD7762 with BSO augmented the growth inhibitory effect of YM155 (Figure 5A, 5B). In both cell lines, there was a reduction in survivin level within 72 h of drug treatment (Figure 5C).

## DISCUSSION

Our model of adaptive resistance was generated by exposing MCF-7, an ER+ BC cell line to escalating doses of YM155. Despite having slower growth rate than drug-naïve P cells, YMR cells escaped very high concentrations of YM155 (Figure 1). Interestingly, the baseline alteration in mitochondrial membrane potential (Figure 1F, right-hand side) that we have observed for YMR cells is unreported so far and requires further exploration.

Increased drug efflux is the most common modes of bypassing drug action. In a recent study by Lamers *et al.*, the MDR1/ATP-binding cassette B1 (ABCB1) was found to be the most differentially expressed gene between YM155-sensitive and -resistant neuroblastoma cell lines [34]. Real-time PCR revealed ABCB1 to be approximately 2.5-fold upregulated, while ABCG2 (another transporter involved in multidrug resistance [35]) was slightly downregulated in YMR cells (Supplementary Figure 3A). However, because of extremely low abundance (Ct value in the range of 33–35) of both mRNA in MCF-7 P and YMR cells, we excluded the possibility of these two molecules to be involved in YM155 resistance. To extend this further, we exposed P and YMR cells to equal amount of YM155 for 1h and performed mass spectrometric analysis to determine drug uptake. No significant difference was found between the amount of drug entered and retained in P versus YMR cells (Supplementary Figure 3B), confirming that adaptation to YM155 in BC cells is unrelated to modification in drug transport mechanisms.

Although, drug adaptation can arise from either alteration in target protein or reduced dependencey on the target for cell survival [19], YM155 continued to inhibit survivin expression in YMR cells to a level similar to that in P cells (Figure 2A–2B). Although, drug withdrawal restored survivin expression (in fact, more than baseline for reason/s not explored here), the resistant phenotype was almost unaffected (Figure 2C). This clearly indicates that firstly, YM155-mediated survivin downregulation is a transient event (particularly important since this drug is administered in 21 d-cycle with each cycle consisting of 7 d drug infusion, followed by 14 d drug-holiday) [12] and secondly, cellular changes imparted by chronic drug exposure are stable, heritable and independent of survivin inhibition.

Bcl-2 family proteins that control intrinsic apoptotic pathway, have also been reported to be downregulated by YM155 [14, 36]. It is possible that chronic drug exposure prompts their upregulation as a compensatory mechanism. However, immunoblot analysis for Bcl-2, Bcl-XL and Mcl-1 demonstrated these proteins to be either downregulated or remain unaltered in YMR cells compared to drug-naïve P cells (Supplementary Figure 4A). Furthermore, ectopic overexpression of survivin in drug-naïve MCF-7 cells did not decrease YM155 sensitivity compared to control cells (Figure 2E). However, genetic ablation of survivin continued to inhibit growth of YM155-sensitive cells (Figure 2H). These strongly implicate that survivin or Bcl-2 proteins are not primary contributors of the growth inhibitory function of YM155.

We speculated genotoxicity of YM155 as its primary mode of action, especially because, previous reports have linked this drug to extensive DNA damage [6, 16]. As an additional support, in a recently published gene expression analysis carried out with three different YM155-sensitive acute lymphoblastic leukemia (ALL) no consistency was observed between survivin downregulation and degree of YM155-sensitivity [37]. However, the genes involved in DNA damage-response were consistently affected by YM155 across those cell lines.

We speculated DNA damage to be contributed by YM155-mediated ROS generation as was reported before [38]. Indeed, this was evident in both MCF-7 P and YMR cell lines (Figure 3A). Furthermore, ROS produced by YMR cells was capable of generating DNA damage in drug-untreated P cells (Supplementary Figure 5). Notably, although persistent drug exposure resulted in higher base-line ROS production in YMR cells, the actual level of damage detected in these cells was lesser than that in P cells (Figure 3B). We assumed this as an adaptive response to long-term drug exposure. In an effort to verify the genotoxic effect of YM155, we looked into the molecules associated with chronic DNA damage-response. One such molecule, phosphorylated H2AX, forms γH2AX foci at the sites of DSB, in an attempt to repair damaged DNA [22]. Again, number of γH2AX foci detected in YMR cells was slightly lower than that in P cells (Figure 3C).

Persistent exposure to genotoxic agents induce senescence-associated growth arrest, TIS [23]. Occasionally, cells undergoing TIS upregulate pro-survival proteins of Bcl-2-family and hence can be inhibited by pharmacological compounds targeting them [39]. Although, YMR cells exhibited multiple evidences of TIS (Figure 3D–3F), obatoclax, a broad spectrum Bcl-2 family inhibitor [40] did not demonstrate greater growth inhibition in YMR cells than P cells (Supplementary Figure 4B). This matches our earlier observation that numerous Bcl-2 family proteins are either unaltered or down, not upregulated in YMR cells (Supplementary Figure 4A).

Based on the reduced amount of DNA damage in YMR cells despite of higher base-line levels of ROS production, we speculated that endogenous anti-oxidants are involved in their detoxification. Therefore, we compared GSH levels between P and YMR cells in presence of YM155. GSH is a tripeptide involved in tumor growth and invasion when exposed to constant level of endogenous or exogenous oxidative stress [28]. Furthermore, increased GSH level is associated with chemotherapy resistance [28]. A gradual increase in GSH level from drug-naïve P cells to 72 h drug-treated P cells to finally in chronically drug-exposed YMR cells (Figure 4A), prompted us to believe that YMR cells endure chronic ROS production by upregulating GSH-dependent anti-oxidant defense system. Confirming this, we reverted the sensitivity of YMR cells to YM155 by exposing them to BSO, the most frequently used molecule to reduce intracellular GSH levels in pre-clinical and clinical studies [28] (Figure 4B, 4C).

Chemotherapy-induced damages to DNA are transmitted to cell cycle check-point proteins, leading to induction of cell cycle arrest and prevention of transfer of damaged DNA to daughter cells [30]. Although, cancer cells have defective check-point mechanisms guiding them to continue neoplastic transformation and progression, pharmaceutical agents exploiting the cell cycle check-points provide a viable anti-cancer strategy [21]. The fact that YMR cells suffer and endure chronic DNA damage, they should be more dependent on cell cycle check-points. Thus, we combined YM155 with AZD7762, an inhibitor of Chk1/2 kinases in order to force the resistant cells to evade the G1 and G2/M check-points, enter mitosis with damaged DNA and ultimately undergo mitotic catastrophe-associated cell death. Indeed, in YMR cells, AZD7762 partly restored YM155 sensitivity (Figure 4D). However, a much superior growth inhibition was achieved in YMR cells treated with a combination of YM155, BSO and AZD7762 than any of these drugs alone (Figure 4E–4G), providing a substantial proof to the notion that proactive mechanisms to combat drug-induced oxidative DNA damage contribute to YM155 adaptation. Therefore, DNA damage is a predominant mechanism of action of this drug. We further confirmed this by treating two drug-naïve (sensitive) ER+ BC cells, T47D and BT474 with YM155, BSO, AZD7762 alone and a combination of three drugs (Figure 5A and 5B). Unlike that in YMR cells, combining AZD, BSO and YM155, does not add onto the growth inhibitory effect of BSO plus YM155 in sensitive cells. Hence, it seems that in sensitive cells blocking cell cycle check-point arrest does not provide extra benefit over the blockage of GSH synthesis for enhancing the anti-tumor activity of the drug. We speculate that persistent DNA-damage has rendered the chronically drug-exposed YMR cells reliant on both anti-oxidant defence and cell cycle check-point arrest mechanisms, while in case of short-term drug exposure of sensitive cells protection from DNA damage can be achieved by raising the anti-oxidant levels alone. Notably, in both of the sensitive cell lines YM155 decreased survivin expression (Figure 5C). Thus, conclusively, genotoxicity is a primary mode of action for YM155 in ER+ BC cells.

A great number of anti-cancer drugs fail in late phase clinical trials. Truly ineffective or abandoned for wrong reasons, either way, attrition of anti-cancer drugs due to late stage failure is unfortunate for patients as well as the drug-development industries. Analogous pattern fits YM155, a survivin-targeting agent with great promise in pre- and early phase clinical trials, but failed in phase II trials. Absence of biomarker-guided patient selection strategy is to be blamed for such disappointing result, which requires a precise knowledge on the mode of drug action. Herein, we have clearly shown that YM155 is a DNA damaging agent capable of producing oxidative stress-induced cytotoxicity in ER-positive BC cells. Considering DNA damage-associated molecules as its predictive biomarkers instead of its intended target survivin and dampening cellular defence mechanisms against YM155-mediated stress response will be key to re-establishing its clinical usefulness.

## MATERIALS AND METHODS

### Cell lines, plasmids, reagents and inhibitors

MCF-7 and T47D, acquired from National Center for Cell Science (Pune, Maharashtra, India), BT474 from Dr. Carlos L. Arteaga (UT Southwestern, Dallas, Texas, USA), were authenticated (Lifecode Technologies Pvt. Ltd., Delhi, India) and grown in DMEM or RPMI-1640 supplemented with 10% fetal bovine serum in a humidified CO_2_ incubator at 37°C. Cell culture reagents were purchased from Thermo Fisher Scientific (Waltham, Massachusetts, USA). Survivin cDNA was amplified from BC cell line and cloned into pEGFP-N1 vector (Clontech Laboratories, Mountain View, California, USA) for developing survivin-overexpressing stable MCF-7 cell line according to standard protocol [41]. Antibodies for survivin, H3K9me3, pChk1 were procured from Cell Signaling Technology (Danvers, Massachusetts, USA) and γH2AX from BioLegend (San Diego, California, USA). PCR Primers were synthesized through Integrated DNA Technologies (Coralville, Iowa, USA). YM155, AZD7762 were purchased from Selleckchem (Houston, Texas, USA) and BSO from Sigma-Aldrich (St. Louis, Missouri, USA). Obatoclax was gifted by Dr. Soumya Sinha Roy (CSIR-Institute of Genomics and Integrative Biology, Delhi, India). Cell culture and imaging glass/plastic-wares were procured from Eppendorf (Hamburg, Germany). Routine chemicals/reagents were purchased from HiMedia Laboratories (Mumbai, Maharashtra, India). Survivin siRNA was purchased from Santa Cruz Biotechnology (Dallas, Texas, USA) and transfected into cells by lipofectamine rnaimax (Thermo Fisher Scientific).

### Measuring cell proliferation/viability

Cell proliferation/viability assays were performed either by counting trypan blue-excluded live cells (proliferation) or measuring ATP levels (viability) with CellTiter-Glo kit (Promega Corporation, Madison, Wisconsin, USA). Colony escape assay (long-term proliferation) was performed by plating cells in low density after 72 h drug/s treatment/s. Cells were stained with 0.5% crystal violet and quantitated once the control well was confluent.

### Immunoblotting and immunofluorescence

For immunoblot analysis, cells were lysed in NP-40 buffer plus phosphatase and protease inhibitor cocktails. Enhanced chemiluminescence-based detection followed by capturing images with the ImageQuant LAS 500 (GE, Boston, Massachusetts, USA) was used.

For live cell imaging, cells were grown in cover glass bottom dishes. Dye/s was added for 15 min. Counterstaining with NucBlue™ (Hoechst 33342) Live ReadyProbes™ reagent (Thermo Fisher Scientific) was done for 10 min. Live cell images at 100× oil objective or mentioned otherwise were captured using Nikon Ti Eclipse inverted microscope (Japan).

For imaging of fixed cells, cells were grown on poly L-lysine (Thermo Fisher Scientific)-coated glass coverslips, fixed with 4% paraformaldehyde and 70% ethanol, permeabilized with triton X-100. Following blocking with 10% BSA, primary antibody incubation was performed. Counterstain was done using NucBlue. Coverslips were mounted on glass slides using anti-fade (n-propyl gallate (Sigma-Aldrich) prepared in DMF and glycerol. Images were captured with Nikon Eclipse Ti-E microscope. Image quantification was done using ImageJ (National Institute of Health, Maryland, USA).

### Real time PCR

RNA was isolated in Trizol (Thermo Fisher Scientific), treated with TURBO DNA-*free*™ Kit (Thermo Fisher Scientific), quantified using NanoDrop (Thermo Fisher Scientific) and used for cDNA synthesis using Superscript III reverse transcriptase (Thermo Fisher Scientific). Real time PCR reaction were performed on BioRad CFX Connect (Bio-Rad Laboratories, Hercules, California, USA) instrument using Sso Fast Eva Green super mix (Bio-Rad Laboratories). Actin was used as house-keeping control. Relative mRNA expression levels were calculated by the standard deltaCt method [42].

### Senescence assay

SA-β-galactosidase staining Kit (Cell Signaling Technology) was used. Imaging was done at 10× in Leica DMi1 (Wetzlar, Germany) and quantified by counting the blue-green cells.

### Comet assay

For detailed protocol see [43]. Briefly, trypsinized cells were mixed with 0.5% Low melting point agarose/LMPA, (Lonza, Basel, Switzerland) at 37°C and plated on pre-coated (1% agarose) glass slides. A sandwich was formed using LMPA on top of this mixture. Cells were lysed in 10 mM Tris-HCl, 2.5 M NaCl, 100 mM EDTA and 1% triton X-100 (v/v) buffer (pH = 10) and incubated in alkaline buffer (300 mM NaOH and 1 mM EDTA, pH > 13), followed by electrophoresis at 21 V/300 mA, neutralization using 400 mM Tris-HCl (pH 7.5) buffer and fixing using absolute ethanol. Staining was done with 1 mg/ml ethidium bromide. Imaging was done using Leica DFC450C microscope (Wetzlar, Germany) at 10× and 40× (inset) magnifications.

### Measurement of mitochondrial membrane potential

Mitochondrial membrane permeabilization was measured with JC-1 dye (10 µM, Thermo Fisher Scientific). A decrease in red: green ratio is indicative of depolarization. Live cell images were captured at 40×.

### ROS detection

ROS was detected using CellROX deep red reagent (5 µM, Thermo Fisher Scientific). Live cell imaging was done at 40×.

### Autophagy assay

Autophagy was detected in live cells by MDC (50 µM, Sigma-Aldrich) staining.

### 3D and sphere assays

For 3D assay, 2500 cells in assay media (DMEM, 2.5% matrigel (Sigma-Aldrich), 5% FBS) were plated in 8 well glass chamber slides pre-coated with matrigel. Mammospheres were grown in sphere media containing DMEM/F12, 10 ng/ml EGF and 1x B-27 (Thermo Fisher Scientific) on 1% LMPA pre-coated 6-well plates at a seeding concentration of 1000 cells/well. Media and drugs were replenished every fourth day. Quantitation was done by dissociating cells with Accutase cell detachment solution followed by trypan blue-exclusion assay.

### GSH assay:

GSH measurement was performed according to published protocol [44] using Ellmans’ reagent (Thermo-Fisher Scientific).

### Statistical analysis

Statistical analyses were performed with GraphPad Prism v5.03 (GraphPad Software, San Diego, CA, USA) (two-tailed, non-parametric *t*-test). Each bar graph is mean of three to four biological replicates along with standard error.

## SUPPLEMENTARY MATERIALS FIGURES



## Abbreviations

BC: breast cancer
YMR: YM155-resistant
P: parental
