# Supplementary material for: Adaptation to chronic exposure to sepantronium bromide (YM155), a prototypical survivin suppressant is due to persistent DNA damage-response in breast cancer cells
Source: Oncotarget. 2018 Sep 11;9(71):33589–600. doi: 10.18632/oncotarget.26096 (PMC6173358; doi:10.18632/oncotarget.26096)
Supplement: Supplementary file 1 [file oncotarget-09-33589-s001.pdf]

Adaptation to chronic exposure to sepantronium bromide (YM155), a prototypical survivin suppressant is due to persistent DNA damage-response in breast cancer cells

SUPPLEMENTARY MATERIALS

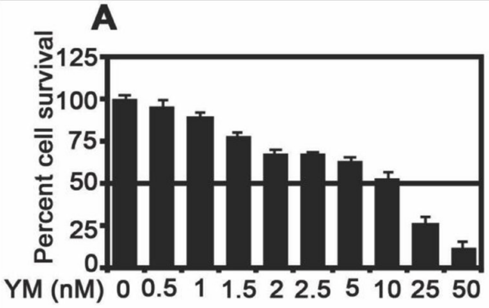

B

	24Hr	48Hr	72Hr
P	10.13	17.54	23.07
YMR	13.46	21.61	26.24

Supplementary Figure 1: Growth characteristics of MCF-7 P and YMR cells. (**A**) Dose response of YM155 on MCF-7 cells as determined by a 72 h CellTiter-Glo assay. (**B**) Doubling time of P versus YMR cells maintained in absence of drug and measured every 24 h interval for up to 72 h. The formula to calculate doubling time is duration*log (2)/log(final cell no.)-log(initial cell no).

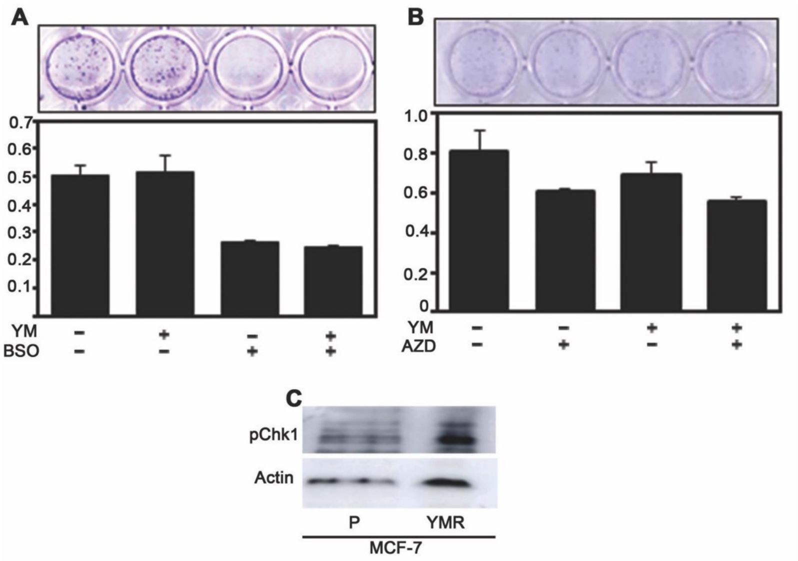

Supplementary Figure 2: YMR cells regain sensitivity to YM155 when combined with BSO or AZD7762. (**A** and **B**) Quantitation of colony escape assay on MCF-7 P versus YMR cells treated with 40 nM YM155 with or without (A) 1 mM BSO and (B) 50 nM AZD7762. (**C**) Immunoblot showing upregulation of pChk1 (S345) following 72h AZD7762 treatment in YMR cells compared to drug-naïve P cells. The ratio of pChk1 intensity between AZD7762-treated P versus YMR cells is 1:1.5 (Image J analysis).

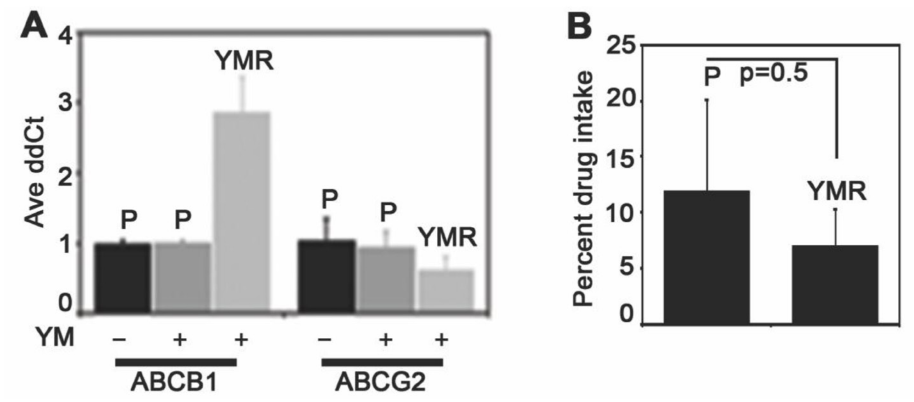

Supplementary Figure 3: YMR cells do not have altered drug transport. (**A**) Real-time PCR comparing mRNA expression levels of ABCB1 and ABCG2 in MCF-7 P versus YMR cells. (**B**) Mass-spectrometry analysis checking YM155 uptake by P versus YMR following 1 h of drug exposure.

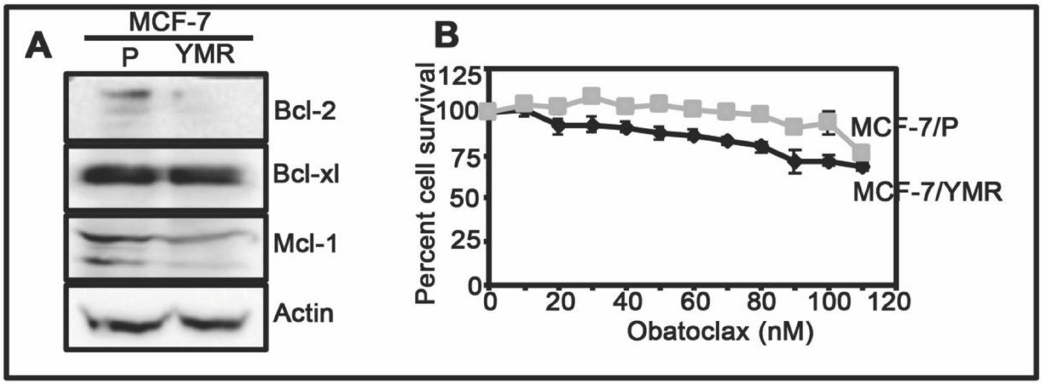

Supplementary Figure 4: YMR cells do not upregulate pro-survival proteins. (**A**) Immunoblot analysis showing levels of Bcl2 family of pro-survival proteins in MCF-7 P and YMR cells. (**B**) CellTiter-Glo assay comparing effect of escalating concentrations of obatoclax, a pan-Bcl2 inhibitor in P versus YMR cells over a 72 h period.

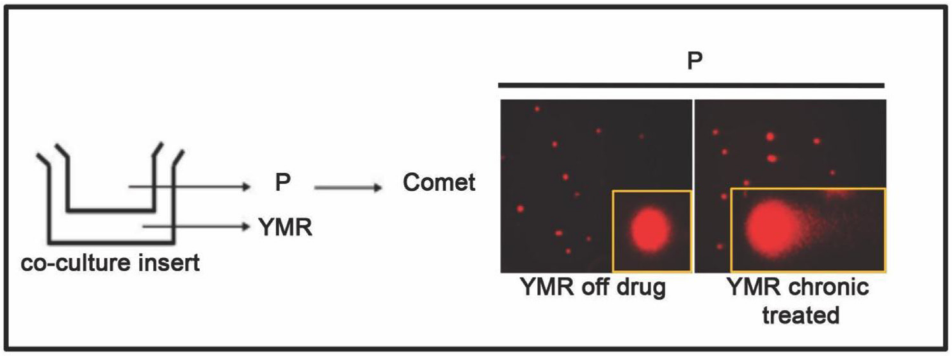

Supplementary Figure 5: ROS produced in YMR cells is diffusible and causes DNA damage in neighboring cells. MCF-7 P and YMR (chronically YM155 treated or not) cells were co-cultured together for 5 h in absence of any drug and comet assay was performed with the P cells.
